# A Longitudinal Study of Retirement Transition and Advance Directive Completion

**DOI:** 10.1093/geroni/igaa057.1424

**Published:** 2020-12-16

**Authors:** Catheryn Koss

**Affiliations:** California State University, Sacramento, Sacramento, California, United States

## Abstract

Advance directives (AD) help to ensure patients’ wishes are honored and contribute to improved end-of-life care. According to normative life course theory, retirement is a significant role change that signals a transition into the third age and its socially prescribed activities. To the extent that ACP is viewed as something to do when one reaches a more advanced stage in life, retirement may spark recognition that planning for incapacity and the end of life is now personally relevant and appropriate. This study tested whether transitioning from work to retirement prompted AD completion. The sample included Health and Retirement Study participants 65 and older who, in 2012, had no ADs and were not completely retired (N = 919). Retirement was operationalized as both a categorical status and as a multistep process. Three waves of data were analyzed using multinomial logistic regression to test associations between retirement transition and advance directive completion. By 2014, 21% had completed ADs and another 17% completed them by 2016. Those who completely retired between 2012 and 2014 were almost twice as likely to complete ADs between 2014 and 2016. Graduated increase in level of retirement between 2012 and 2014 was associated with higher odds of new AD possession in 2016, but did not reach statistical significance at p < .05. These results suggest the period following retirement may be an optimal time to encourage patients and clients who have not already done so to complete advance directives.

